# Molecular mechanisms underlying drug resistance in protozoan parasites: emerging mechanisms and therapeutic perspectives

**DOI:** 10.3389/fpara.2026.1934996

**Published:** 2026-08-26

**Authors:** Kavithma Uduwela, Koshila Ranasinghe

**Affiliations:** Department of Zoology and Environmental Management, Faculty of Science, University of Kelaniya, Kelaniya, Sri Lanka

**Keywords:** antiprotozoal therapy, drug resistance, genomic plasticity, molecular mechanisms, protozoan parasites

## Abstract

Protozoan parasitic infections, including malaria, leishmaniasis, and human African trypanosomiasis, remain major global public health challenges. In the absence of highly effective vaccines, disease control relies primarily on chemotherapy; however, the emergence and spread of drug-resistant parasite populations increasingly threaten treatment efficacy. This review synthesizes current evidence on the molecular mechanisms underlying drug resistance in *Plasmodium*, *Leishmania*, and *Trypanosoma* species through a systematic analysis of literature. The review identifies four interconnected mechanisms that drive the evolution of drug resistance. First, altered drug transport enables parasites to regulate intracellular drug concentrations through mutations, loss, or amplification of membrane transporters, including PfCRT in *Plasmodium* and AQP2 in *Trypanosoma brucei*. Second, target modification and genomic plasticity promote resistance through point mutations in drug targets, such as *dhfr* and *dhps* in *Plasmodium*, while kinetoplastids, particularly *Leishmania*, exploit extensive genomic plasticity, including aneuploidy, gene amplification, and translational reprogramming, to facilitate rapid adaptation under drug pressure. Third, metabolic reprogramming enhances parasite survival by increasing intracellular thiol production, strengthening antioxidant defense systems, and reshaping central carbon and lipid metabolism to mitigate drug-induced stress. Finally, stress response and persistence mechanisms enable subpopulations of parasites to enter dormant, persister-like states characterized by reduced metabolic activity and slowed proliferation, thereby evading both host immune responses and chemotherapeutic agents. Collectively, these findings demonstrate that drug resistance is a dynamic, multifactorial evolutionary process rather than a single molecular event. Addressing this growing challenge requires integrating genomic surveillance, molecular diagnostics, mathematical modeling of resistance transmission, and mechanistic insights into parasite persistence into future drug discovery and disease control strategies. Such an integrated approach is essential for improving the durability of antiprotozoal therapies and advancing global efforts to control neglected protozoan diseases.

## 1 Introduction

Protozoan parasitic infections remain one of the most significant challenges to global public health, disproportionately affecting populations in *tropica*l and sub*tropica*l regions. Diseases such as malaria (*Plasmodium* spp.), leishmaniasis (*Leishmania* spp.), Human African Trypanosomiasis (*Trypanosoma brucei*), and Chagas disease *(Trypanosoma cruzi)* account for substantial morbidity and mortality worldwide (Tarannum et al., 2023). Despite combined elimination efforts, the control of these pathogens relies heavily on chemotherapy due to the absence of effective vaccines for most protozoan diseases. While antimicrobial agents have historically been successful in reducing parasite burdens, their efficacy is increasingly threatened by the emergence and spread of drug-resistant strains (Capela et al., 2019).

**Figure 1:**
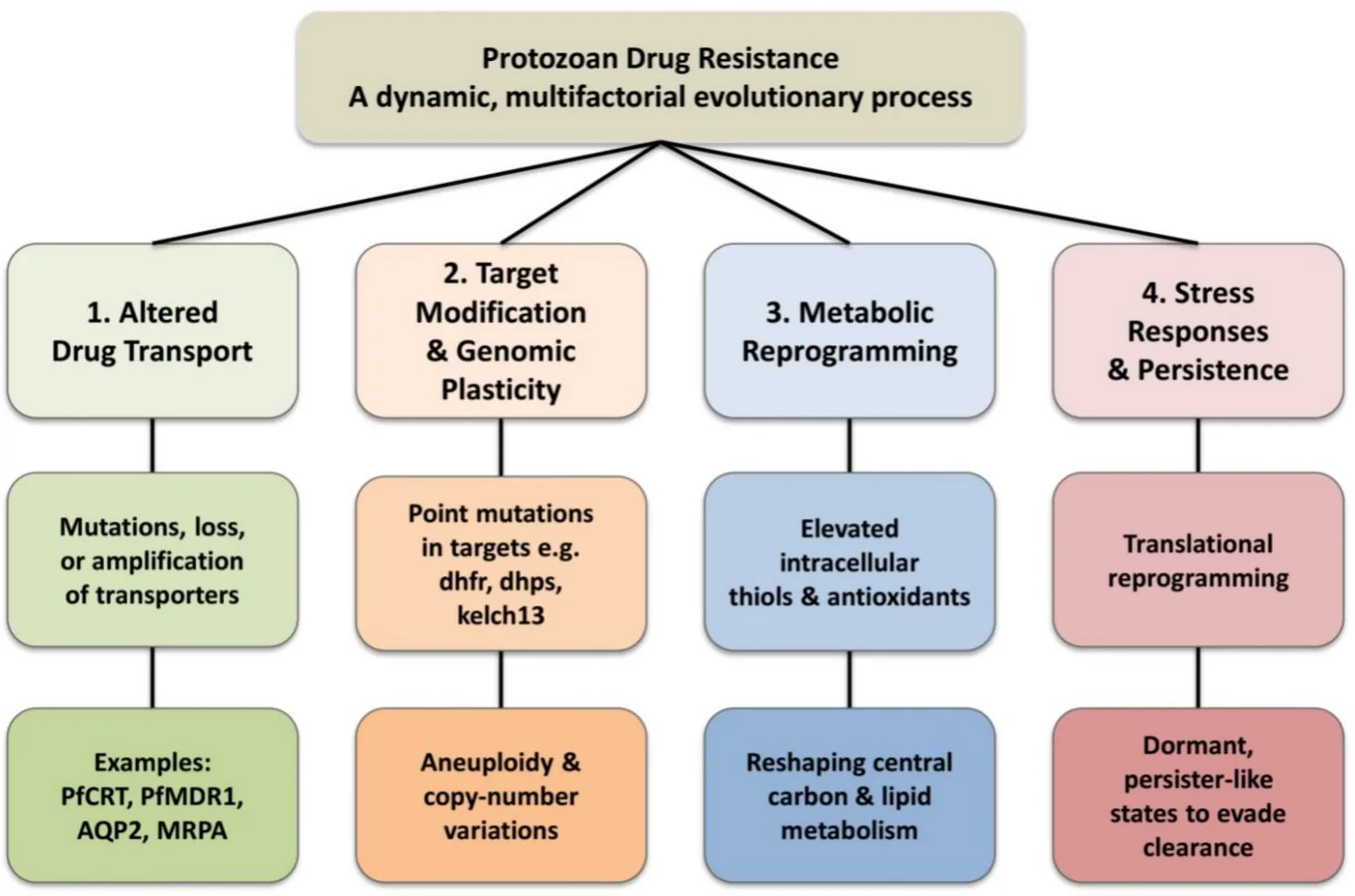
Conceptual overview of resistance mechanisms (author-made).

The molecular landscape of drug resistance in these parasites is multi-factorial and evolutionary dynamic (**Figure 1**). In *Plasmodium falciparum*, resistance to artemisinin-based combination therapies (ACTs) and partner drugs is driven by specific genetic polymorphisms (Rocamora and Winzeler, 2020; Wicht et al., 2020). Recent genomic surveillance utilizing Targeted Amplicon Deep Sequencing (TADS) has identified high prevalence rates of mutations in the *dhfr* and *dhps* genes, which confer resistance to sulfadoxine-pyrimethamine (L’Episcopia et al., 2023). Furthermore, alterations in the PfCRT (chloroquine resistance transporter) and PfMDR1 (multidrug resistance 1) proteins modulate the transport of 4-aminoquinoline drugs away from their target sites in the digestive vacuole (L’Episcopia et al., 2023; Wicht et al., 2020). While mutations in the Kelch13 (K13) propeller domain, which is the primary marker for artemisinin partial resistance, remain rare in some African populations, the potential for their expansion necessitates urgent monitoring (L’Episcopia et al., 2023; Wicht et al., 2020).

Beyond malaria, kinetoplastid parasites exhibit unique resistance mechanisms that challenge standard pharmacotherapy. In *Trypanosoma brucei*, resistance to arsenicals like melarsoprol has been definitively linked to the loss of the aquaglyceroporin 2 (AQP2) transporter, rendering the parasite unable to take up the drug (Fairlamb and Horn, 2018). Similarly, *Leishmania* species have developed sophisticated methods to survive exposure to antimonials, miltefosine, and amphotericin B (Ponte-Sucre et al., 2017). Recent research identifies translational reprogramming as a major driver of this resistance, arranging coordinated changes in the parasite’s metabolome and lipidome to support survival under stress (Moncada-Diaz et al., 2024). This capacity for adaptation is further complicated by the phenomenon of ‘persisters’, which are parasite sub-populations that enter a state of dormancy or quiescence to evade both immune clearance and drug toxicity, eventually leading to disease recurrence (Tarannum et al., 2023).

In addition to transport alterations and target modifications, metabolic reprogramming serves as a crucial evolutionary strategy for parasite survival under chemotherapeutic stress (Capela et al., 2019). Parasites frequently upregulate detoxification pathways and alter central carbon and lipid metabolism to mitigate drug-induced oxidative damage and maintain redox homeostasis (Tarannum et al., 2023). For instance, Leishmania species exhibit enhanced intracellular thiol production, such as trypanothione, which facilitates the sequestration and neutralization of antimonial drugs prior to them reaching their intended targets (Ponte-Sucre et al., 2017). Similarly, for nitroheterocyclic drugs used against *Trypanosoma*, resistance is often achieved by altering the specific nitroreductase enzymes required to activate the prodrugs, blurring the lines between metabolic adaptation and target modification (Capela et al., 2019). This metabolic plasticity acts in concert with other resistance mechanisms, providing a resilient background layer that lowers the effective potency of treatments and facilitates the selection of high-level resistance alleles (Capela et al., 2019; Tarannum et al., 2023).

Addressing these challenges requires a paradigm shift in how drug resistance is integrated into public health and development pipelines. It is no longer sufficient to treat resistance merely as a clinical endpoint. It must be incorporated into the earliest stages of drug discovery (Hefnawy et al., 2016). This includes confirming the efficacy of novel compounds against recent clinical isolates and utilizing pharmacokinetic/pharmacodynamic (PK/PD) mathematical modeling to predict population-level transmission outcomes (Hefnawy et al., 2016; Slater et al., 2016).

Furthermore, the ability to elucidate these complex resistance mechanisms is increasingly dependent on the evolution of modern genomic methodologies (Rocamora and Winzeler, 2020). The transition from traditional phenotypic assays to Next-Generation Sequencing (NGS) has revolutionized the genomic surveillance of protozoan parasites (Rocamora and Winzeler, 2020). NGS provides the critical depth of coverage needed to detect low-frequency resistant subclones within mixed infections before they become clinically dominant (Tandoh et al., 2026). It also offers the resolution necessary to decode the complex genomic architectures of kinetoplastids, where resistance is frequently driven by aneuploidy and copy-number variations rather than simple nucleotide substitutions (Laffitte et al., 2016). However, the application of these advanced sequencing technologies requires overcoming significant bioinformatic challenges. These bioinformatic challenges include navigating the extreme AT-rich compositional biases of *Plasmodium* genomes (Hamilton et al., 2016), resolving the dynamic structural variations in *Leishmania* (Laffitte et al., 2016), and integrating multi-omics data to accurately map post-transcriptional and translational reprogramming under chemotherapeutic stress (Chaparro et al., 2020; Moncada-Diaz et al., 2024).

In light of these rapid evolutionary adaptations, it is imperative to adopt forward-looking strategies in antiprotozoal drug discovery. Consequently, this review will also explore future directions in the field, highlighting the necessary shift toward targeting parasite-specific proteins that lack human homologues to minimize host toxicity. Promising examples include calcium-dependent protein kinases (CDPKs) in apicomplexans (Sharma et al., 2021) and parasite-derived secreted kinases in kinetoplastids (Silvestre et al., 2022). Crucial to this next generation of drug development is the rigorous validation of these targets using advanced genetic engineering tools like CRISPR/Cas9 (Lander and Chiurillo, 2019). Finally, this review will discuss the transition toward ethically sound translational platforms, such as 3D bioengineered tissues, organoids, and humanized mice, which provide superior, human-relevant alternatives to conventional animal models for evaluating novel chemotherapies (Ehret et al., 2017). By integrating advanced genomic surveillance with these novel drug discovery paradigms, the global health community can better anticipate and counter the dynamic threat of protozoan drug resistance.

This literature review aims to synthesize current knowledge regarding these molecular mechanisms. It will examine the genetic underpinnings of resistance in *Plasmodium, Leishmania* and *Trypanosoma*, analyze the role of genomic surveillance in guiding treatment policies (L’Episcopia et al., 2023; Mvumbi et al., 2015; Rocamora and Winzeler, 2020), and discuss the necessity of bridging the gap between molecular biology and rational drug design to combat the neglect of these diseases.

## 2 Methodology

The objective of this literature review was to identify publications describing the molecular mechanisms underlying drug resistance in protozoan parasites, with a focus on *Plasmodium, Leishmania*, and *Trypanosoma* species. The electronic databases PubMed, Google Scholar, Scopus, and Web of Science were searched for articles. Search terms combined general concepts of protozoan drug resistance with parasite-specific and mechanistic keywords, using Boolean operators. Examples of search strings included “protozoan drug resistance”, “molecular mechanisms of drug resistance”, “drug resistance in protozoa” AND (*Plasmodium* OR *Leishmania* OR *Trypanosoma*), and parasite-specific combinations such as “*Plasmodium falciparum* AND molecular basis of drug resistance”, “*Leishmania* AND antimonial resistance AND transporter”, and “*Trypanosoma brucei* AND melarsoprol resistance AND aquaglyceroporin”

The inclusion-exclusion criteria were used when selecting the relevant literature for the analysis. Studies were included if they were peer-reviewed journal articles or reviews in English, focused on human-infecting Plasmodium, Leishmania, or *Trypanosoma* species, and provided molecular or biochemical insights into drug resistance. Eligible mechanisms included changes in drug uptake or efflux (ex: transporters and membrane proteins), drug target modifications (ex: point mutations, gene amplification, altered isoforms), genomic and transcriptomic alterations, metabolic adaptation and detoxification pathways, and stress response or persistence mechanisms.

Studies were excluded if they were non-peer-reviewed sources, non-English, or if they focused primarily on clinical outcomes, epidemiology, or public-health policy without describing molecular mechanisms. Articles dealing exclusively with non-protozoan pathogens (bacteria, viruses, fungi) or protozoa outside the three focused genera were also excluded, except where they provided brief comparative context for conserved strategies. Additional relevant articles were identified by screening the reference lists of key primary studies and recent reviews on drug resistance in *Plasmodium, Leishmania*, and *Trypanosoma.*

## 3 Literature analysis

### 3.1 Altered drug uptake and efflux

Altered drug uptake and efflux are core, early events in protozoan drug resistance, because changes in membrane transport systems let parasites keep intracellular drug levels below toxic thresholds despite adequate plasma exposure. By modulating influx and efflux, parasites separate the pharmacokinetics seen by the host from the concentrations that reach intracellular targets, so treatment can fail even when dosing is correct. In *Plasmodium*, *Leishmania* and *Trypanosoma*, this is achieved through modifications of solute carriers, aquaglyceroporins, and ATP- binding cassette (ABC) transporters acting at the plasma membrane or internal compartments such as the digestive vacuole (Wicht et al., 2020).

In *Plasmodium falciparum*, chloroquine resistance mediated by the digestive- vacuole transporter PfCRT is the most characteristic example (Wicht et al., 2020). Wild- type parasites accumulate chloroquine in the acidic vacuole, where the drug interferes with haem detoxification, but resistance- associated PfCRT isoforms allow efflux of protonated chloroquine back into the cytosol, lowering local concentrations at the site of action and permitting continued hemoglobin digestion. PfMDR1, a P- glycoprotein–like transporter on the same membrane, further shapes susceptibility to chloroquine, mefloquine and artemisinin- based combination therapy (ACT) partner drugs by influencing their partitioning between cytosol and vacuole in an allele- specific method. Field data from Benin illustrate how these transporters track changes in front- line therapy (Wicht et al., 2020). More than a decade after ACT deployment, Pfcrt K76 wild- type alleles again predominate, consistent with re- emerging chloroquine sensitivity, while Pfmdr1 N86 and Y184F, linked to reduced lumefantrine susceptibility, which are frequent in artemether–lumefantrine–treated populations (L’Episcopia et al., 2023). This shift suggests that selection has moved from favoring chloroquine- resistant configurations towards variants that preserve ACT efficacy yet subtly reduce partner- drug accumulation.

**Figure 2:**
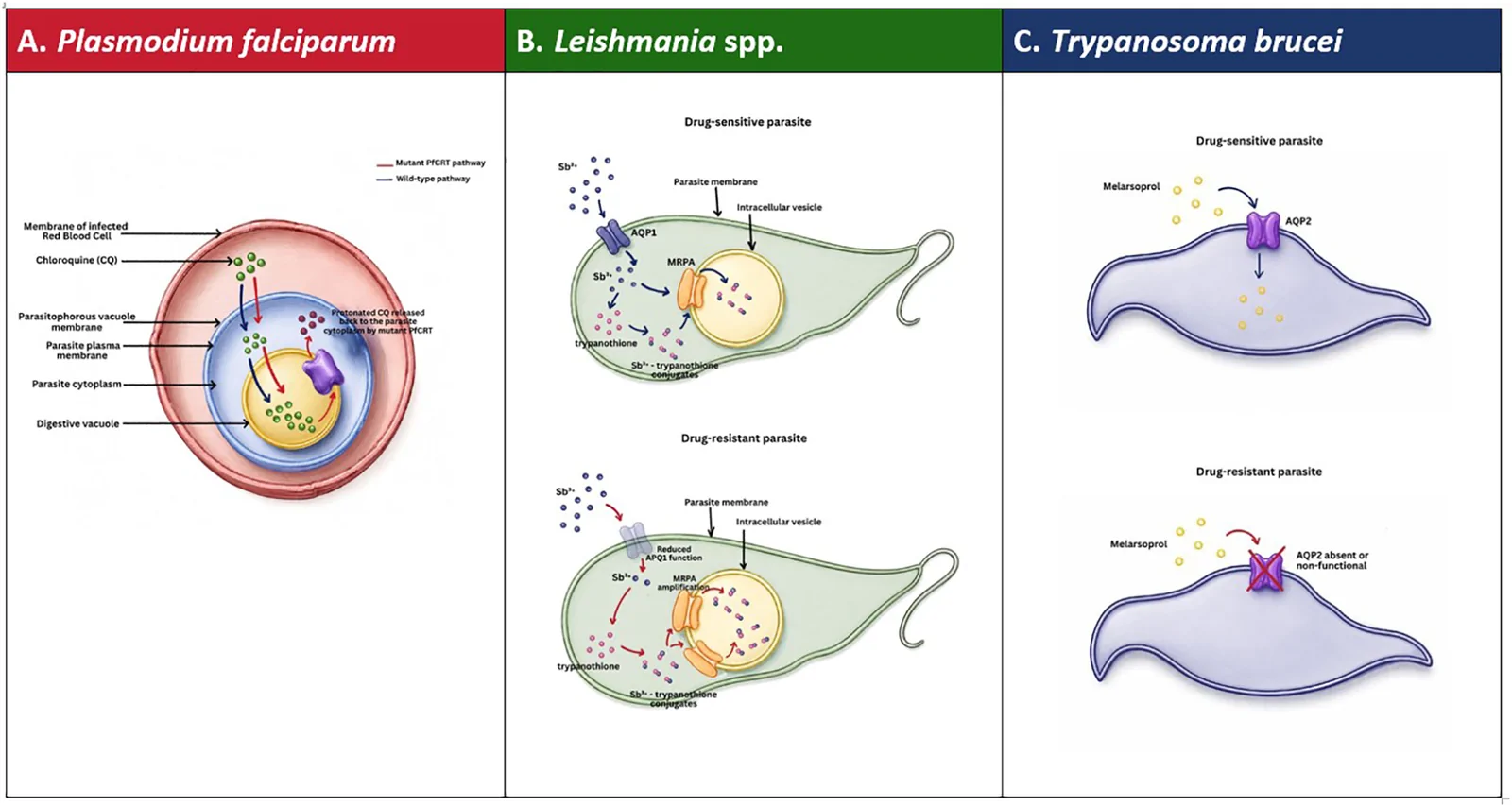
Subcellular localization and function of key drug transporters implicated in resistance across *Plasmodium*, *Leishmania*, and *Trypanosoma* species. **(A)** In *Plasmodium falciparum*, depicted within a red blood cell and bounded by the parasitophorous vacuole membrane (PVM), transporters like PfCRT pump drugs out of the acidic digestive vacuole into the parasite cytosol, lowering drug concentration at the target site. **(B)** In *Leishmania* spp., altered transporters reduce the influx of drugs like antimonials and miltefosine, or sequester them into vesicles via MRPA. **(C)** In *Trypanosoma brucei*, the aquaglyceroporin AQP2 normally mediates the influx of drugs; its deletion or loss of function blocks drug entry, conferring resistance to melarsoprol and pentamidine (Author-made).

Additional *P. falciparum* transporters contribute to multidrug efflux (Capela et al., 2019). PfMRP1 and related ABC transporters at the parasite plasma membrane export organic anions, glutathione conjugates and several antimalarials. Genetic disruption of PfMRP1 increases sensitivity to chloroquine, quinine and artemisinin, indicating an important protective role. These surface pumps likely cooperate with digestive- vacuole transporters such as PfCRT and PfMDR1, broadening the range of metabolites and drugs that can be removed or redistributed and creating redundancy that makes transporter- based resistance robust and evolutionarily attractive. Population genomic surveys repeatedly detect polymorphisms and copy- number variation in pfmdr1, pfmrp and other transporter loci in parasites under drug pressure, underscoring altered transport as a front- line adaptation (Capela et al., 2019) (**Figure 2**).

In *Leishmania* spp., uptake and efflux of antimonials and miltefosine provide a well- studied paradigm for transporter- driven resistance. Pentavalent antimonials (SbV) require reduction to SbIII to become active, and SbIII enters parasites mainly via the aquaglyceroporin AQP1. Resistant isolates commonly show reduced AQP1 expression, inactivating mutations or deletions encompassing the AQP1 gene, all of which decrease SbIII influx (Ponte-Sucre et al., 2017). At the same time, the ABC transporter MRPA, located on vesicles near the flagellar pocket, sequesters SbIII–trypanothione conjugates into an intracellular compartment, effectively removing the active drug from cytosolic targets. MRPA amplification via episomes or chromosome 23 polysomy, together with elevated trypanothione synthesis, substantially enhances this sequestration and is strongly associated with high- grade antimonial resistance on the Indian subcontinent. The combination of reduced uptake through AQP1 and increased sequestration/efflux via MRPA and other ABC transporters illustrates how *Leishmania* constructs multi- layered barriers to drug entry and retention (Ponte-Sucre et al., 2017).

Miltefosine resistance follows a parallel but distinct route centred on the miltefosine transporter complex LdMT/LdRos3. Miltefosine, a phospholipid analogue, is imported by this P- type ATPase-flippase system, and *in vitro* selection repeatedly yields LdMT or LdRos3 point mutations, truncations and subtelomeric deletions that markedly reduce drug internalisation (Hefnawy et al., 2016). Resistant promastigotes show more than 95% reductions in miltefosine uptake, accompanied by shifts in fatty- acid chain length, degree of unsaturation, and ergosterol content, indicating that altered membrane composition reinforces decreased transporter activity by further limiting drug partitioning into parasite membranes. Aneuploidy and local copy- number changes at chromosomal regions bearing LdMT and AQP1 can further tune transporter dosage, providing “many roads to drug resistance” in which distinct genomic lesions converge on similar low- uptake phenotypes (Hefnawy et al., 2016). Recent work in antimony- resistant *Leishmania tropica* shows that translational reprogramming also co- ordinates expression of transporters, lipid- metabolism enzymes and efflux pumps, adding a post- transcriptional layer of control over uptake and efflux systems (Moncada-Diaz et al., 2024) (**Figure 2**).

Kinetoplastids causing African trypanosomiasis to provide a particularly clear example of how loss of a non- essential uptake route can generate high- impact clinical resistance (Fairlamb and Horn, 2018). In *Trypanosoma brucei*, the aquaglyceroporin AQP2, localised to the flagellar pocket, mediates uptake of melarsoprol and pentamidine, even though these drugs are larger and more complex than classical aquaglyceroporin substrates. Comparative genomics of laboratory- selected and clinical isolates reveals that melarsoprol- and pentamidine- resistant strains frequently harbour complete deletions of AQP2 or chimeric AQP2/3 genes created by recombination in the tandem AQP2–AQP3 locus, disrupting the pore properties required for drug transport (Fairlamb and Horn, 2018). Parasites lacking AQP2, often along with the P2 aminopurine transporter AT1, remain fully viable *in vitro* and in rodent infections and are transmissible via tsetse flies, indicating that this drug- uptake pathway is largely dispensable for parasite survival. Such dispensability makes AQP2 an easy target for loss- of- function mutations under drug pressure and explains the rapid spread of melarsoprol resistance despite its potency (**Figure 2**).

The altered transport theme extends to broader efflux systems and host and parasite interfaces. In *P. falciparum*, additional ABC transporters likely collaborate to export glutathione conjugates, haem- related metabolites and drugs, while in *Leishmania*, multiple ABC proteins (including ABCI4, ABCG2 and PRP1) can efflux metal and thiol complexes and xenobiotics, further lowering effective intracellular drug levels (Capela et al., 2019; Ponte-Sucre et al., 2017). Surface glycoconjugate changes in antimony- resistant *Leishmania donovani* drive IL- 10 up- regulation and MDR1 overexpression in host macrophages, increasing host- cell efflux of antimonials and indirectly reducing parasite drug exposure, a striking example of resistance achieved partly through manipulation of host transport pathways (Ponte-Sucre et al., 2017).

Overall, these observations support altered uptake and efflux as foundational resistance mechanisms that arise early and interact with target modification, metabolic rewiring and stress responses. Because many key transporters such as PfCRT, PfMDR1, AQP1, AQP2, LdMT, and several ABC proteins are non- essential or functionally redundant, protozoa can tolerate large changes in their activity or even complete loss, gaining resistance with modest fitness costs in the absence of drug (Capela et al., 2019; Fairlamb and Horn, 2018; Hefnawy et al., 2016). This highlights the vulnerability of drugs that rely on single, dispensable entry routes and underscores the value of designing compounds that enter via multiple or essential pathways or access compartments less exposed to classic resistance transporters (Fairlamb and Horn, 2018). Key genetic markers associated with drug resistance across the major protozoan parasites are summarized in **Table 1**.

**Table 1:** Key genetic markers of drug resistance by parasite species (author-made).

Parasite species	Drug/treatment	Primary mechanism of resistance	Specific genetic markers/targets	References
*Plasmodium falciparum*	Sulfadoxine- pyrimethamine	Target modification	*dhfr* (N511, C59R, S108N) and *dhps* (A437G, K540E, A581G) mutations.	L'Episcopia et al., 2023
*Plasmodium falciparum*	Artemisinin (ACTs)	Target modification/ Stress handling	*kelch13* (pfk13) mutations; *pfcoronin* mutations.	L'Episcopia et al., 2023; Wicht et al., 2020
*Leishmania spp.*	Amphotericin B	Target modification/ Genomic Plasticity	Altered sterol C24- methyltransferase and C5-desaturase (CYP51).	Ponte-Sucre et al., 2017
*Leishmania donovani*	Miltefosine	Altered uptake/ Reduced internalisation	LdMT/LdRos3 mutations and subtelomeric deletions.	Hefnawy et al., 2016
*Trypanosoma brucei*	Melarsoprol	Altered uptake/ Loss of function	AQP2 deletion or chimeric AQP2/3 genes.	Fairlamb and Horn, 2018

### 3.2 Drug target modifications and genomic plasticity

Drug target modification and broader genomic plasticity form a second major axis of protozoan drug resistance that complements altered uptake and efflux. Rather than only lowering intracellular drug levels, parasites change the structure or abundance of the molecules that drugs bind to, so inhibition is reduced while essential functions are maintained. In *Plasmodium*, this usually involves point mutations and copy- number changes in well- defined enzymatic or transporter targets, whereas in *Leishmania* and other kinetoplastids such as *Trypanosoma*, it often relies on aneuploidy, gene amplification and multi- gene adaptations that reshape entire pathways (L’Episcopia et al., 2023).

In *Plasmodium falciparum*, resistance to antifolate drugs (sulfadoxine–pyrimethamine and related combinations) is the classic example of target- site modification. Antifolates inhibit dihydrofolate reductase (DHFR) and dihydropteroate synthase (DHPS), and stepwise accumulation of mutations in dhfr (N51I, C59R, S108N) and dhps (A437G, K540E, A581G, A613S) progressively reduces drug binding while preserving folate metabolism. Molecular surveillance in Benin illustrates this process: targeted amplicon deep sequencing revealed high frequencies of the dhfr triple mutant N51I/C59R/S108N and the dhps A437G mutation, with a substantial proportion of parasites carrying triple or quintuple dhfr/dhps haplotypes predictive of sulfadoxine-pyrimethamine treatment failure (L’Episcopia et al., 2023). However, the absence of dhps K540E and low prevalence of A581G indicate that the highest- level resistance genotypes (RII/RIII) remain rare, so SP still retains some utility for intermittent preventive treatment, emphasizing how specific mutation combinations define clinically relevant resistance thresholds.

Artemisinin resistance adds further complexity because it involves both a defined primary marker and multiple modifying loci (White and Chotivanich, 2024). In Southeast Asia, delayed parasite clearance and increased ring- stage survival after artemisinin treatment are strongly associated with mutations in the kelch13 (pfk13) propeller domain, and gene- editing experiments have validated these alleles as causal drivers of reduced artemisinin susceptibility. Genomic studies show that pfk13- mutant lineages carry distinct Haplotypes that have undergone strong positive selection, consistent with repeated emergence and spread of resistance alleles under ACT pressure. Yet *in vitro* evolution and genome- wide association have revealed pfk13- independent routes to artemisinin tolerance, including mutations in pfcoronin, copy- number variants in stress- response genes on chromosome 14, and changes in pfap2mu and pfubp1 that regulate endocytosis and protein trafficking, all converging on pathways that modulate haemoglobin uptake and stress handling. These findings show that even when a single marker gene is useful for surveillance, the resistance phenotype can be genetically heterogeneous and multigeneric (White and Chotivanich, 2024).

Target modification in *Plasmodium* also affects candidate compounds in development (Rao et al., 2023). *In vitro* and *in vivo* evolution against dihydroorotate dehydrogenase (DHODH) inhibitors such as DSM265 has repeatedly produced missense mutations and gene amplifications in PfDHODH, and clinical trials have already detected emergent PfDHODH variants in patients failing DSM265. These studies indicate that drug target genes often have sufficient mutational and dosage flexibility to accommodate resistance without catastrophic loss of function, highlighting the need to anticipate target- site evolution during preclinical development. On the other hand, the inability to select resistance *in vitro* to some fast- killing or host- targeting compounds suggests that not all targets are equally permissive, and that “low mutational capacity” targets may provide more durable options (Rao et al., 2023).

In *Leishmania*, target modification is tightly intertwined with extreme genome plasticity (Laffitte et al., 2016). Parasites frequently alter not only protein sequences but also chromosome copy number and local gene dosage, producing polygenic resistance phenotypes (Laffitte et al., 2016). Amphotericin B (AmB), which kills *Leishmania* by binding ergosterol- like sterols and forming membrane pores, is a clear example (Ponte-Sucre et al., 2017). AmB- resistant isolates show profound remodeling of sterol biosynthesis, including decreased ergosterol and accumulation of alternative intermediates such as cholesta- 5, 7, 24- trien- 3β- ol, reducing the drug’s preferred binding sites in the membrane (Ponte-Sucre et al., 2017). These changes arise from mutations and copy- number alterations in sterol- pathway enzymes (sterol C24- methyltransferase, C5-desaturase (CYP51) and are often accompanied by broader lipidome shifts that help maintain membrane integrity despite altered sterol content (Ponte-Sucre et al., 2017). Because ergosterol biosynthesis is essential, parasites must balance lowering AmB binding with preserving sterol function, resulting in complex multi- gene resistance architectures rather than simple single- point mutations (Laffitte et al., 2016; Ponte-Sucre et al., 2017).

Genomic and transcriptomic work on miltefosine resistance in *Leishmania donovani* likewise shows that resistance extends beyond the primary miltefosine transporter complex (Hefnawy et al., 2016). Mutations in pyridoxal kinase and an α- adaptin- like protein, together with differential expression of genes involved in stress responses, membrane composition and folate metabolism, suggest that the drug’s effective target landscape spans several pathways (Hefnawy et al., 2016). Whole- genome sequencing of clinical and experimental lines demonstrates that aneuploidy is adaptive and environment- dependent: chromosomes carrying virulence or resistance genes can become polysomic under *in vitro* selection, then revert towards disomy in mammalian hosts, implying that target- related amplifications may arise and disappear dynamically with drug exposure (Downing et al., 2011; Laffitte et al., 2016). This fluidity complicates efforts to link specific structural changes to resistance but also highlights how *Leishmania* uses gene- dosage modulation as a rapid, reversible way to adjust effective target levels (Laffitte et al., 2016).

Beyond structural and dosage changes, translational reprogramming acts as a powerful layer of “functional” target modification in *Leishmania* (Moncada-Diaz et al., 2024). Because these parasites rely heavily on post- transcriptional control, drug- resistant *L. tropica* lines display extensive differences in the set of mRNAs that are actively translated compared with sensitive lines, even without drug (Moncada-Diaz et al., 2024). Ribosome profiling has identified thousands of differentially translated transcripts in antimony- resistant parasites, including those encoding enzymes of trypanothione metabolism, lipid biosynthesis and redox regulation, indicating that the effective pool of protein targets and resistance modulators is reshaped at the translation level (Chaparro et al., 2020; Moncada-Diaz et al., 2024). This “pre- emptive” translational rewiring allows parasites to maintain a proteome pre- optimised for survival under drug challenge, lowering the vulnerability of multiple pathways without necessarily changing coding sequences (Moncada-Diaz et al., 2024).

In kinetoplastids such as *Trypanosoma brucei*, target- site changes are described for some drugs, though the best- known genetic determinant of resistance (AQP2) is a transporter rather than a classical enzymatic target (Baker et al., 2012; Fairlamb and Horn, 2018). For nitroheterocyclic drugs (nifurtimox, benznidazole), resistance in trypanosomes and *T. cruzi* is often linked to loss or reduced activity of nitroreductases that activate the prodrugs, making the activation enzyme the effective target rather than the ultimate molecular lesion (Capela et al., 2019). Resistance to eflornithine, which is an inhibitor of ornithine decarboxylase, can involve mutations or down regulation of the amino- acid transporter AAT6 that imports the drug (Capela et al., 2019). It can again blur the distinction between target and transport adaptation (Capela et al., 2019). Altogether, drug- target modification and genomic plasticity provide protozoa with a flexible repertoire of resistance options that complement transporter- based mechanisms and challenge efforts to rely on single molecular markers (Capela et al., 2019; Laffitte et al., 2016).

### 3.3 Metabolic adaptation and detoxification pathways

Metabolic reprogramming allows protozoan parasites to buffer the outcome of target inhibition and oxidative damage induced by chemotherapy. In *Leishmania*, elevated levels of intracellular thiols such as trypanothione, glutathione, and cysteine are repeatedly observed in antimony- resistant strains, where they facilitate sequestration of antimony-thiol conjugates into intracellular vesicles via MRPA (Multi-drug Resistance-associated Protein A) and enhance detoxification of reactive oxygen species (Ponte-Sucre et al., 2017). Overexpression of enzymes in the trypanothione synthesis pathway, as well as tryparedoxin peroxidase, further strengthens antioxidant defenses and contributes to a multifactorial resistance phenotype that encompasses both parasite- intrinsic and macrophage- mediated changes (Ponte-Sucre et al., 2017). Genomic and transcriptomic analyses suggest that these metabolic shifts are tightly linked to genome plasticity, with aneuploidy and gene amplifications adjusting the dosage of key metabolic genes in response to drug pressure (Moncada-Diaz et al., 2024).

Metabolic plasticity is also important in kinetoplastid persistence under drug exposure. Studies of *Trypanosoma cruzi* amastigotes and *Trypanosoma brucei* bloodstream forms indicate that adaptation of central carbon metabolism, mitochondrial function and redox balance can support survival in the face of nitroheterocyclic drugs and host- imposed stresses, often in slow- growing or dormant sub-populations that are more tolerant to chemotherapy (Tarannum et al., 2023; Capela et al., 2019). These adaptations may include increased reliance on alternative energy pathways, changes in lipid metabolism, and modulation of antioxidant systems, although the exact nodes differ between species and developmental stages (Tarannum et al., 2023).

In *Plasmodium falciparum*, metabolic contributions to resistance are less thoroughly mapped than transporter and target mutations, but genomic approaches have implicated genes involved in stress responses, protein turnover and redox homeostasis as potential modifiers of drug sensitivity (Capela et al., 2019; Rocamora and Winzeler, 2020). For instance, recent examples demonstrate that resistance to compounds such as fosmidomycin and halofuginone is linked to adaptive metabolic responses, including significant alterations in proline metabolism (Cowell et al., 2018; Rocamora and Winzeler, 2020). Collectively, the available data suggest that metabolic adaptation acts as an important “background” layer that can lower the effective potency of drugs and facilitate the selection and maintenance of high- level resistance alleles in parasite populations (Capela et al., 2019; Tarannum et al., 2023).

### 3.4 Stress responses, translational reprogramming and persistence

Stress-response mechanisms and phenotypic persistence add a further protective layer against antiparasitic drugs that operates independently of, yet in coordination with, fixed genetic resistance. In *Leishmania*, recent evidence indicates that alterations in translational output play a central role in shaping resistance to antimonials and miltefosine. Resistant *Leishmania tropica* lines exhibit widespread changes in ribosome engagement across the transcriptome relative to susceptible parasites, and notably, these differences are maintained even without drug pressure (Moncada-Diaz et al., 2024). Such translational shifts are accompanied by coordinated remodeling of metabolic and lipid pathways that enhance survival under chemotherapeutic stress. These adaptive programs act alongside chromosomal and copy-number changes, reinforcing the idea that post-transcriptional control, rather than transcriptional regulation alone, is a primary determinant of gene expression outcomes in *Trypanosoma*tids (Moncada-Diaz et al., 2024). Modulators of this process, including calcium-dependent protein kinase CDPK1, can selectively influence the translation of mRNAs encoding transporters and metabolic enzymes, further linking translational regulation to the emergence and maintenance of drug resistance (Moncada-Diaz et al., 2024).

Closely related to these adaptive responses is the phenomenon of parasite persistence, which centers on small subpopulations capable of surviving otherwise lethal drug exposure and subsequently re-establishing infection. Comparative analyses across protozoan parasites indicate that *Plasmodium*, *Trypanosoma* and *Leishmania* can all enter persister-like states in response to environmental or pharmacological stress (Tarannum et al., 2023). These states are typically marked by slowed or arrested growth, reduced metabolic activity, condensed chromatin, and extensive reorganization of both transcriptomes and translatomes. In *Plasmodium falciparum*, brief exposure to dihydroartemisinin enriches for dormant or inactive ring-stage parasites that can resume proliferation days after treatment cessation, a phenotype that is particularly noticeable in parasites harboring artemisinin-resistant *kelch13* variants and is associated with adaptations of mitochondrial and apicoplast function (Tarannum et al., 2023). In contrast, *Plasmodium vivax* hypnozoites exemplify long-term persistence, displaying expression signatures dominated by RNA-binding proteins and translational repressors, underscoring the importance of post-transcriptional regulation in sustaining prolonged dormancy (Tarannum et al., 2023).

Persister-like behaviors are also evident among kinetoplastids. Dormant or slow-replicating *Trypanosoma cruzi* amastigotes and tissue-resident *Trypanosoma brucei* forms are increasingly recognized as contributors to chronic infection and inconsistent therapeutic efficacy (Cook et al., 2023; Tarannum et al., 2023). In bloodstream trypanosomes, immune evasion mechanisms such as the variant surface glycoprotein coat and complement-interacting receptors, including ISG65, reduce host-mediated clearance, thereby maintaining niches in which drug-tolerant parasites can persist (Cook et al., 2023; Fairlamb and Horn, 2018). Taken together, these observations support a model in which persistence, translational reprogramming and stress-induced cellular remodeling constitute core elements of the drug-resistant phenotype itself, rather than secondary consequences of classical resistance mutations (Tarannum et al., 2023; Moncada-Diaz et al., 2024).

### 3.5 Emerging mechanisms of drug resistance

Recent studies have revealed that protozoan parasites employ additional mechanisms of drug resistance that extend beyond classical pathways such as altered drug transport, target modification, metabolic adaptation, and stress-response pathways. These emerging mechanisms involve mutations in non-essential genes, dysregulation of vesicular trafficking pathways, and translational and post-translational modifications, highlighting the dynamic and multifaceted nature of parasite adaptation to chemotherapeutic pressure (Alsford et al., 2012; Dumetz et al., 2018).

#### 3.5.1 Loss-of-function mutations in non-essential genes

Drug resistance frequently arises through loss-of-function mutations in genes that are not the direct targets of the drug, provided those genes are dispensable for parasite survival under normal conditions (Cowell et al., 2018). When a drug relies on a specific, non-essential cellular pathway for uptake, activation, or intracellular trafficking, parasites can simply jettison that pathway to evade toxicity (Dumetz et al., 2018).

Aquaglyceroporin 2 (AQP2) in *Trypanosoma brucei* is the most definitive experimentally supported example is the loss of the AQP2 transporter (Baker et al., 2012). AQP2 normally resides in the flagellar pocket and is responsible for the influx of the arsenical drug melarsoprol and the diamidine pentamidine (Munday et al., 2013). Because AQP2 is not strictly required for the parasite’s viability in the mammalian host or for transmission via the tsetse fly, parasites can easily tolerate complete deletions, nonsense mutations, or the creation of chimeric, non-functional AQP2/3 genes (Fairlamb and Horn, 2018). The loss of this non-essential gene severely restricts intracellular drug accumulation, driving high-level clinical resistance (Baker et al., 2012; Munday et al., 2013).

Transporters in *Leishmania* spp. Is a parallel mechanism occurs with antimonial and miltefosine resistance. The aquaglyceroporin AQP1 (which mediates the influx of active trivalent antimony) and the LdMT/LdRos3 transporter complex (responsible for miltefosine internalization) can undergo inactivating point mutations, truncations, or subtelomeric deletions under drug pressure (Hefnawy et al., 2016; Ponte-Sucre et al., 2017). Because these uptake pathways exhibit functional redundancy or are not strictly vital for basal survival, the parasite sacrifices them to prevent lethal drug accumulation (Hefnawy et al., 2016).

#### 3.5.2 Vesicular trafficking pathways and drug response

Vesicular trafficking has emerged as a critical and dynamic determinant of drug susceptibility in protozoan parasites (Dumetz et al., 2018). Cellular processes such as endocytosis, vesicle transport, and membrane remodeling regulate the uptake, intracellular distribution, and sequestration of both essential nutrients and therapeutic compounds (Alsford et al., 2012). Alterations in these pathways provide the parasite with unique, multi-layered mechanisms to evade drug action:

Artemisinin and Haemoglobin Uptake in *Plasmodium* is a premier example of altered trafficking conferring resistance is seen with artemisinin-based therapies (Wicht et al., 2020). Artemisinin is a prodrug that requires activation by free heme, which is generated when the parasite digests host red blood cell hemoglobin in its digestive vacuole. Resistance, often mediated by mutations in the *Kelch13* propeller domain or the adaptor protein complex *pfap2mu*, which results in a significant reduction in the endocytosis of host cell cytoplasm (Rocamora and Winzeler, 2020; Wicht et al., 2020). This diminished hemoglobin uptake leads to reduced hemoglobin digestion and, consequently, a lower pool of free heme. With less heme available, artemisinin activation is severely decreased, allowing the parasite to survive the short-lived drug exposure in a persistent, dormant-like state (Wicht et al., 2020).

Ganaplacide (KAF156) and Intracellular Trafficking: Another highly relevant example involves GNF179, whose clinical analogue KAF156 (ganaplacide) has advanced into clinical trials as a next-generation antimalarial (Rao et al., 2023). Resistance to this class of compounds is frequently associated with mutations in genes governing intracellular trafficking and membrane dynamics, such as the *Plasmodium falciparum* cyclic amine resistance locus (PfCARL), as well as vesicle-associated proteins like *PfUbp1* (Cowell et al., 2018; Rocamora and Winzeler, 2020). Disruption or alteration of these trafficking-related processes prevents the drug from successfully reaching its intracellular target or alters the localization of essential membrane proteins, demonstrating that rewiring vesicular transport is a robust, alternative strategy for parasite survival (Cowell et al., 2018).

#### 3.5.3 Translational and post-translational regulation

Beyond genetic mutations, parasites can adapt to drug pressure through dynamic changes in translational and post-translational regulatory mechanisms (Cowell et al., 2018). Because protozoa, particularly kinetoplastids, often rely heavily on post-transcriptional control rather than transcriptional initiation, modulating translation efficiency and protein stability provides a rapid and reversible strategy for surviving chemotherapeutic stress (Moncada-Diaz et al., 2024).

Pre-emptive Translational rewiring in *Leishmania* is one recent example. Recent translatomic studies utilizing ribosome profiling in antimony-resistant *Leishmania tropica* have revealed massive shifts in the pool of actively translated mRNAs, even in the absence of drug pressure (Moncada-Diaz et al., 2024). Transcripts encoding enzymes vital for trypanothione metabolism, redox regulation, and lipid biosynthesis are selectively loaded onto ribosomes, creating a proteome “pre-optimised” to handle drug-induced oxidative stress without requiring changes to the underlying coding sequences (Moncada-Diaz et al., 2024). Modulators such as the calcium-dependent protein kinase CDPK1 and specific translation initiation factors (e.g., eIF4A) are implicated in orchestrating this selective mRNA translation (Chaparro et al., 2020; Moncada-Diaz et al., 2024).

RNA-Binding Proteins and *Plasmodium* Dormancy occurs when translational regulation is equally critical for the survival of persister stages, which evade drug toxicity by entering prolonged states of metabolic dormancy (Tarannum et al., 2023). For example, the persistence of *Plasmodium vivax* hypnozoites is characterized by unique expression signatures heavily dominated by RNA-binding proteins and translational repressors (Tarannum et al., 2023). These regulatory proteins likely pause the translation of bulk metabolic machinery while preserving essential mRNAs, allowing the parasite to safely weather extended drug exposure and re-establish infection once treatment ceases (Tarannum et al., 2023).

Post-Translational Protein Turnover, once proteins are synthesized, post-translational modifications and targeted degradation pathways tightly control their abundance and function (Alsford et al., 2012). Ubiquitin-mediated proteolysis and altered protein turnover rates enable parasites to rapidly clear damaged proteins caused by drug stress, or to selectively downregulate drug transporters to minimize compound uptake (Alsford et al., 2012; Cowell et al., 2018). In high-throughput chemogenomic screens, disruptions in these protein homeostasis networks often directly influence drug sensitivity, underscoring their role as an emerging resistance node (Cowell et al., 2018).

Collectively, these emerging mechanisms demonstrate that protozoan drug resistance extends beyond classical genetic alterations and involves complex interactions between intracellular trafficking, gene regulation, and protein homeostasis (Dumetz et al., 2018). Understanding these pathways will be crucial for identifying novel therapeutic targets and developing strategies to overcome resistance in parasitic diseases (Dumetz et al., 2018).

### 3.6 Cross-parasite themes, surveillance gaps and implications for drug development

Comparing *Plasmodium, Leishmania* and *Trypanosoma* reveals recurring mechanistic themes alongside lineage- specific strategies. Across all three parasite groups, resistance commonly emerges through a combination of altered drug uptake or efflux, changes to drug targets, metabolic reconfiguration and activation of stress-response pathways. However, the balance between these mechanisms differs markedly, particularly with respect to the roles of genome plasticity, post-transcriptional control, and the contribution of persistent parasite stages (Capela et al., 2019). In *Plasmodium falciparum*, resistance phenotypes are frequently driven by discrete point mutations in a relatively small set of loci, including pfcrt, pfmdr1, dhfr/dhps, and kelch13. By contrast, *Leishmania* parasites more often exploit large-scale genomic rearrangements such as copy-number variation and aneuploidy, coupled with extensive regulation at the level of translation. *Trypanosoma brucei* provides a distinct example in which disruption of a single, non-redundant drug-entry pathway, most notably loss of the aquaglyceroporin AQP2, can dramatically reduce drug efficacy and translate directly into clinical failure (Fairlamb and Horn, 2018; Moncada-Diaz et al., 2024; Ponte-Sucre et al., 2017).

Despite major advances in defining these molecular mechanisms, their application to resistance surveillance and drug development remains limited. In many endemic settings, systematic monitoring of established resistance markers is still lacking, and experimental validation of resistance-associated loci emerging from genomic studies remains uneven, particularly for parasites other than *Plasmodium falciparum* and *Leishmania donovani* (Capela et al., 2019; Hefnawy et al., 2016; Rocamora and Winzeler, 2020). In malaria research, modelling studies have emphasized the need to couple pharmacokinetic and pharmacodynamic measurements with transmission dynamics to better predict how drug properties and resistance pathways will influence control and elimination efforts, an issue of growing importance as artemisinin-based combinations are increasingly deployed for chemoprevention and mass drug administration (Slater et al., 2016). In the context of leishmaniasis, there have been calls to incorporate resistant parasite lines and *in vitro* resistance-selection experiments directly into early-stage drug discovery, ensuring that candidate compounds are screened against plausible future resistance scenarios (Hefnawy et al., 2016; Ponte-Sucre et al., 2017; Cohen and Azas, 2021). Given the evident ability of protozoan parasites to repeatedly evolve diverse, and sometimes convergent, molecular responses to drug pressure, successful next-generation therapies will likely need to account for transporter redundancy, genome flexibility and persistence-related tolerance from the outset to maximize their durability (Capela et al., 2019; Tarannum et al., 2023).

## 4 Genomic methodologies and detection of drug resistance

The elucidation of the molecular mechanisms driving drug resistance are ranging from transporter mutations to extensive genomic plasticity, which relies fundamentally on the evolution of sequencing technologies (Rocamora and Winzeler, 2020). While traditional parasitology relied heavily on *in vitro* sensitivity assays and phenotypic characterization, the contemporary gold standard for resistance surveillance is molecular diagnostics.

### 4.1 The shift from sanger to next-genration sequencing

Historically, Sanger sequencing provided the foundational data for identifying point mutations in key drug targets, such as the *dhfr* and *dhps* genes in *Plasmodium falciparum* (L’Episcopia et al., 2023). However, Sanger sequencing is fundamentally limited by its inability to detect minor allele frequencies in mixed infections, a common occurrence in high-transmission endemic regions (Tandoh et al., 2026).

Next-Generation sequencing (NGS) has revolutionized the genomic surveillance of protozoa parasites (Rocamora and Winzeler, 2020). By enabling massively parallel sequencing, NGS provides the depth of coverage required to detect low-frequency resistant subclones before they become clinically dominant, making credible variant calls more efficiently (Tandoh et al., 2026). Furthermore, NGS is essential for resolving the complex genomic architectures seen in kinetoplastids, where drug resistance is often mediated by aneuploidy, copy-number variations (CNV) and gene amplification rather than simple nucleotide substitutions (Laffitte et al., 2016).

### 4.2 Bioinformatic challenges in parasite genomics

The application if Next-Generation Sequencing technologies is heavily reliant on advanced bioinformatics, as assembling and interpreting protozoan genomes presents a unique set of computational hurdles. Standard genomic pipelines designed for mammalian or bacterial genomes often fail to accurately capture the structural complexities and extreme biases inherent to these parasites.

#### 4.2.1 Compositional bias and amplification errors in plasmodium

The genome of *Plasmodium falciparum* is notoriously AT-rich, with an overall AT content exceeding 80%, and reaching up to 90% in non-coding and telomeric regions (Hamilton et al., 2016). This extreme base composition poses significant challenges during the PCR amplification steps of sequencing library preparation, often resulting in severe sequencing bias. Consequently, downstream bioinformatic analysis frequently encounters uneven read coverage across the genome. High-AT regions are prone to misalignment and exhibit a high rate of small indel mutations relative to other species, necessitating specialized mapping algorithms and high-depth coverage to ensure accurate identification of target-site modifications (Hamilton et al., 2016; Rocamora and Winzeler, 2020).

#### 4.2.2 Quantifying genomic plasticity in kinetoplastids

For kinetoplastids like Leishmania, the primary bioinformatic challenge lies in resolving large-scale structural variations. Drug resistance in these organisms is frequently mediated by gene amplification, aneuploidy, and complex multi-gene adaptations rather than simple point mutations (Laffitte et al., 2016; Ponte-Sucre et al., 2017). Standard short-read sequencing often struggles to span highly repetitive regions, making it difficult to assemble the complete chromosomal architecture. Bioinformatic pipelines must account for the dynamic nature of these genomes. Differentiating between transient, environment-dependent polysomy, which may revert to disomy once the parasite enters a mammalian host and fixed genetic adaptations requires comparative longitudinal genomic analysis (Laffitte et al., 2016). Additionally, tracking the unbalanced dosage changes in aneuploid chromosomes requires multi-omics integration to understand broad-scale disruptions in transcript and protein stoichiometry (Cuypers et al., 2022).

#### 4.2.3 Analyzing post-transcriptional and translational reprogramming

Beyond the DNA sequence, protozoan drug resistance involves profound shifts in gene expression. Because kinetoplastids rely heavily on post-transcriptional control, bioinformatic analysis must extend to transcriptomics and translatomics (Moncada-Diaz et al., 2024). Analyzing ribosome profiling data (polysome-profiling) to detect translational reprogramming requires mapping protected mRNA fragments to open reading frames (Chaparro et al., 2020). Integrating these complex translatomic datasets with genomic profiles is a computational bottleneck that must be overcome to fully map the multi-layered defense systems these parasites employ under chemotherapeutic stress (Chaparro et al., 2020; Moncada-Diaz et al., 2024).

## 5 Future directions

The rapid evolution of drug resistance in protozoan parasites necessitates a forward-looking approach to drug discovery. To prevent future treatment failures, researchers must not only identify novel therapeutic targets but also adopt modern, ethically sound frameworks for validating these targets and testing drug efficacy.

### 5.1 Novel drug pipelines and parasite-specific targets

Traditional antiprotozoal drugs have often been hindered by high toxicity and severe side effects due to the biochemical similarities between eukaryotic parasites and their human hosts. Consequently, modern drug discovery is shifting towards targeting parasite-specific proteins that lack human homologues.

In apicomplexan parasites like Plasmodium spp., calcium-dependent protein kinases (CDPKs) represent highly promising targets. These protozoa rely on CDPKs to sense and transduce calcium signals at critical stages of their development, facilitating vital processes such as host cell invasion, translational regulation, and transmission between the human host and vector (Moncada-Diaz et al., 2024; Sharma et al., 2021). Because human cells utilize different biochemical pathways for calcium signaling, drugs designed to inhibit protozoan CDPK1 could provide potent antimalarial effects with minimal host toxicity. However, many compounds currently progressing through the drug discovery pipeline target a much broader range of pathways. Expanding beyond CDPKs, research is heavily focused on other essential kinases, such as Phosphatidylinositol 4-kinase (PI4K), as well as targeting parasite-specific metabolic nodes and lipid biosynthesis pathways across different species (Sharma et al., 2021).

Similarly, in kinetoplastids such as Leishmania and *Trypanosoma*, parasite-derived kinases that are released directly into the host cell are being investigated as novel targets to circumvent fixed genetic resistance mechanisms (Silvestre et al., 2022). These kinases manipulate host immune responses and facilitate intracellular survival, making them critical nodes for therapeutic intervention (Silvestre et al., 2022).

### 5.2 Genetic engineering and target validation via CRISPR-Cas9

Before investing in the costly pharmaceutical development of inhibitors, candidate drug targets and resistance-associated loci must be rigorously validated (Capela et al., 2019). Modern target identification frequently relies on robust strategies such as *in vitro* drug resistance selection combined with mass spectrometry based methods to map altered proteomes and pinpoint candidate proteins. Notably, many targets identified through these strategies have advanced to clinical trials, with CRISPR-Cas9 playing a critical role in their downstream functional validation. The adaptation of Clustered Regularly Inter-spaced Short Palindromic Repeats (CRISPR)/Cas9 technology has revolutionized this target validation process in protozoan parasitology (Lander and Chiurillo, 2019).

Historically, kinetoplastids like *Trypanosoma cruzi* and *Leishmania* spp. were notoriously difficult to manipulate genetically. However, CRISPR/Cas9 systems now allow researchers to efficiently induce targeted double-strand breaks to perform precise gene knockouts, endogenous tagging, and gene replacement (Iwanaga et al., 2022; Lander and Chiurillo, 2019). By utilizing Eukaryotic Pathogen CRISPR/Cas9 design tools, scientists can down regulate specific gene families, such as those encoding putative fatty acid transporters or critical drug entry routes like AQP2, to observe phenotypic changes and directly validate whether the loss or modification of a specific gene renders the parasite resistant or completely non-viable (Fairlamb and Horn, 2018; Lander and Chiurillo, 2019).

### 5.3 Ethical paradigms and alternatives to animal models

Historically, understanding host-pathogen interactions and evaluating drug efficacy required extensive use of *in vivo* rodent models. However, standard laboratory mice often present significant limitations; the anatomical, immunological, and genetic differences between mice and humans can result in vast discrepancies in disease progression and drug response, acting as severe confounders in translational research (Ehret et al., 2017). Furthermore, the reliance on animal models is increasingly restricted by rigorous ethical constraints.

To bypass these hurdles, researchers are transitioning to advanced *in vitro* and 3D bioengineering platforms. Technologies such as primary human cell cultures, organoids, and microfluidic “organ-on-a-chip” devices can replicate the physiological complexity of human tissues far more accurately than standard 2D cultures. Additionally, where animal hosts remain strictly necessary, researchers are utilizing highly specialized transgenic “humanized” mice, which are manipulated to express components of the human immune system, thereby bridging the translational gap while minimizing overall animal usage (Ehret et al., 2017). These alternatives provide precise, controlled environments for testing novel chemotherapies against persistent or active parasite stages while adhering to modern ethical imperatives (Tarannum et al., 2023).

## 6 Conclusions

Drug resistance in protozoan parasites emerges from a complex, dynamic interplay of molecular mechanisms that collectively undermine the efficacy of the limited chemotherapeutic options available for malaria, leishmaniasis, and trypanosomiasis (Blasco et al., 2017; Croft et al., 2006). This review highlights that resistance is rarely a single genetic event. Instead, parasites construct multi-layered defenses: they exploit non-essential membrane transporters to alter drug efflux jettison dispensable uptake pathways entirely (e.g., PfCRT, AQP2, and MRPA), utilize highly mutable targets like *dhfr* and *kelch13*, and rely heavily on extreme genomic plasticity and aneuploidy to adjust gene dosage (Ariey et al., 2013; Baker et al., 2012). Furthermore, emerging mechanisms such as the rewiring of vesicular trafficking and endocytosis not only drive resistance to front-line artemisinins but also threaten next-generation antimalarials like ganaplacide (Cowell et al., 2018; Dumetz et al., 2018). Beyond structural genomic changes, parasites utilize pre-emptive translational reprogramming, RNA-binding proteins, and targeted protein degradation to maintain proteostasis under drug pressure (Alsford et al., 2012; Moncada-Diaz et al., 2024). These metabolic reprogramming and post-transcriptional translational shifts allow subpopulations to enter dormant, persister-like states, evading both immune clearance and pharmacological stress entirely (Haldar et al., 2018).

Understanding and combating these evolutionary adaptations is increasingly reliant on advanced genomic methodologies. The transition to Next-Generation Sequencing (NGS) has provided unprecedented resolution to track low-frequency resistant subclones and map large-scale chromosomal rearrangements (Cowell and Winzeler, 2019; Miotto et al., 2015). However, maximizing the utility of these tools requires overcoming significant bioinformatic bottlenecks, particularly in resolving the extreme AT-rich biases of Plasmodium and the environment-dependent polysomy characteristic of Leishmania (Downing et al., 2011).

Moving forward, the global health community must shift from reactive clinical surveillance to proactive, integrated drug discovery. This necessitates targeting parasite-specific nodes, such as calcium-dependent protein kinases (CDPKs), which minimize human host toxicity (White, 2004). It also requires the rigorous, early-stage validation of these targets using CRISPR/Cas9 genetic engineering (Moore, 2015), and the ethical evaluation of novel compounds using advanced 3D bioengineered tissues and humanized models rather than traditional rodent assays. By integrating pharmacokinetic/pharmacodynamic (PK/PD) mathematical modeling with advanced molecular surveillance, researchers can better predict resistance selection windows and extend the durability of both existing and next-generation antiprotozoal therapies (Dondorp et al., 2009; Menard and Dondorp, 2017).
